# The Pathological G51D Mutation in Alpha-Synuclein Oligomers Confers Distinct Structural Attributes and Cellular Toxicity

**DOI:** 10.3390/molecules27041293

**Published:** 2022-02-15

**Authors:** Catherine K. Xu, Marta Castellana-Cruz, Serene W. Chen, Zhen Du, Georg Meisl, Aviad Levin, Benedetta Mannini, Laura S. Itzhaki, Tuomas P. J. Knowles, Christopher M. Dobson, Nunilo Cremades, Janet R. Kumita

**Affiliations:** 1 Yusuf Hamied Department of Chemistry, University of Cambridge, Lensfield Road, Cambridge CB2 1EW, UK; catherine.xu@mpl.mpg.de (C.K.X.); mc2033@cam.ac.uk (M.C.-C.); gm373@cam.ac.uk (G.M.); al680@cam.ac.uk (A.L.); bm475@cam.ac.uk (B.M.); tpjk2@cam.ac.uk (T.P.J.K.); 2 Department of Life Sciences, South Kensington Campus, Imperial College London, London SW7 2AZ, UK; weiyan89@gmail.com; 3 Department of Pharmacology, University of Cambridge, Tennis Court Road, Cambridge CB2 1PD, UK; zhen_du2020@163.com (Z.D.); lsi10@cam.ac.uk (L.S.I.); 4 Cavendish Laboratory, Department of Physics, University of Cambridge, JJ Thomson Avenue, Cambridge CB3 0HE, UK; 5 Institute for Biocomputation and Physics of Complex Systems (BIFI), University of Zaragoza, Mariano Esquillor, Edificio I+D+I, 50018 Zaragoza, Spain

**Keywords:** α-synuclein, toxic oligomers, Parkinson’s disease, familial mutations, α-helical structure

## Abstract

A wide variety of oligomeric structures are formed during the aggregation of proteins associated with neurodegenerative diseases. Such soluble oligomers are believed to be key toxic species in the related disorders; therefore, identification of the structural determinants of toxicity is of upmost importance. Here, we analysed toxic oligomers of α-synuclein and its pathological variants in order to identify structural features that could be related to toxicity and found a novel structural polymorphism within G51D oligomers. These G51D oligomers can adopt a variety of β-sheet-rich structures with differing degrees of α-helical content, and the helical structural content of these oligomers correlates with the level of induced cellular dysfunction in SH-SY5Y cells. This structure–function relationship observed in α-synuclein oligomers thus presents the α-helical structure as another potential structural determinant that may be linked with cellular toxicity in amyloid-related proteins.

## 1. Introduction

The misfolding of proteins and their aggregation into amyloid fibrils has been implicated in numerous neurodegenerative disorders, including Parkinson’s disease (PD) and Alzheimer’s disease (AD) [1]. In PD, aggregates of the 14 kDa protein α-synuclein are the major component of Lewy bodies and neurites, which emerge as the pathological hallmarks of the disease. In solution, α-synuclein is intrinsically disordered; however, upon interaction with membranes, the protein has been observed to adopt an α-helical structure [2], associated with the functional role of the protein in neuronal cells [3]. In addition to the random coil to α-helix transition upon membrane binding, α-synuclein can also adopt a β-sheet structure upon self-assembly into amyloid aggregates, a process in which membranes might also play a role [4,5,6]. Oligomeric species with varying degrees of β-sheet structure are observable in the early stages of aggregation [7]. It is these early oligomeric species, rather than the mature amyloid fibrils, that are believed to be key toxic species in the context of disease [1,8,9,10,11]. While a range of mechanisms have been proposed by which the toxic effect could be mediated, interactions with cell membranes are likely to be a main contributor to the observed cytotoxicity [12,13,14].

Duplications and triplications of the WT α-synuclein gene, and a number of single-point mutations, are associated with familial cases of PD, which present with both earlier onset and faster progression of the disease [15]. The causative relationship between an increased load of the protein, which results from the duplication or triplication of the gene, and earlier onset of the disease is likely to simply be a consequence of the increased aggregation propensity of α-synuclein due to its increased concentration. By contrast, the aetiology of the familial cases associated with the pathological variants remains unknown and a variety of mechanisms and reasons for the connection of these mutations to disease have been proposed [16,17,18,19,20,21,22,23,24].

All familial PD-associated mutations identified thus far are located in the N-terminal region of α-synuclein (Figure S1A) and have been suggested to alter the membrane binding properties and thus the function of α-synuclein [19,25,26,27,28,29,30,31,32,33,34]. However, no clear associations with protein dysfunction have been established yet. While the A30P and G51D mutations have been reported to abrogate α-synuclein–membrane interactions, the E46K variant may enhance membrane binding [25,26,27,29,32,35]. Furthermore, studies on the in vitro aggregation kinetics of these α-synuclein variants have yielded conflicting results [15,18,19,20,21,22,23,24,36,37,38,39]. While the E46K, H50Q, and A53T variants have been found to aggregate more rapidly than WT, G51D exhibits a lower propensity for aggregation, and A30P appears to be the most variable in behaviour. Nevertheless, it is clear that aggregation kinetics and membrane binding are insufficient to explain the link between these mutations and disease.

Given that intermediate oligomeric species are proposed to be a major source of toxicity, several studies have sought to characterise the effects of familial PD-associated mutations on α-synuclein oligomers [40,41,42]. Paslawski et al. used hydrogen/deuterium exchange mass spectrometry to study oligomers of WT α-synuclein and the A30P, E46K, and A53T variants, finding very subtle differences between deuterium exchange profiles [43]. Furthermore, through single-molecule FRET (smFRET) experiments on the same variants, the concentrations of oligomers produced in aggregation reactions were found to be the same for all variants, indicating that structural differences between variant oligomers are likely to have a more significant pathological effect than simply levels of oligomers [44]. Moreover, this study also identified differences in the intermolecular FRET efficiencies between variant oligomers, again demonstrating that these mutations affect oligomer structure.

Detailed structural characterisation of oligomers generated in situ in aggregation reactions is highly challenging due to their heterogeneous and transient nature, whereas the use of stable kinetically trapped model systems allows us to obtain in-depth structural and biological information on the nature of these species [13,43]. Here, we characterise the effects of familial PD-associated mutations on α-synuclein oligomers, and reveal a distinct α-helical structural polymorphism within the G51D oligomers that correlates with increased cellular dysfunction in SH-SY5Y cells.

## 2. Results and Discussion

### 2.1. All α-Synuclein Variants Form Oligomers with Similar Size and Morphology

**Figure 1:**
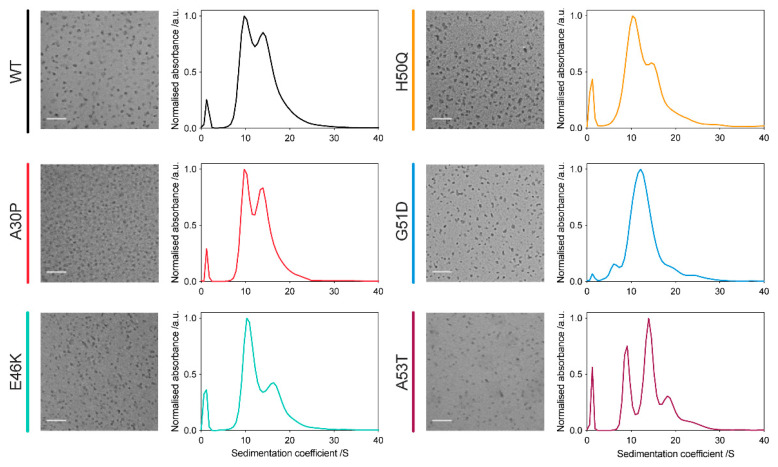
All α-synuclein variants form oligomers with similar size and morphology. TEM images (**left** panels) of variant oligomers, confirming that relatively homogeneous oligomer populations are produced in all cases, with a roughly spherical shape and 5–15 nm diameter (scale bar = 100 nm). AUC analysis of variant oligomers (**right** panels), demonstrating the size distributions of oligomers within each sample, with the peak at 2S arising due to the residual monomeric protein present in the oligomeric samples.

Oligomers from the familial PD-associated α-synuclein variants were successfully generated using our previously described protocols [45]. The oligomers were characterised using transmission electron microscopy (TEM), which showed that these variant oligomers have a similar size and overall morphology to the WT oligomers, being approximately spherical with a diameter of around 5–15 nm, consistent with dynamic light scattering analysis, which showed a clear size distinction between those of the monomers and oligomers (Figure 1, left panels; Figure S2). Detailed investigation of the oligomer size distributions by analytical ultracentrifugation (AUC) sedimentation velocity analysis and native-PAGE demonstrated conserved stable sizes for the oligomers, with all variant oligomers, except for the G51D variant, containing oligomer populations at both 10S and 15S, in different proportions (Figure 1, right panels; Figure S1B). While the A53T variant contained an additional population at 19S, and a lowly populated species at 24S, suggesting the presence of larger aggregate species for this variant, the distribution of G51D oligomers showed just one major population around 12S, suggesting that there may be interactions that restrict the preferred sizes of such oligomers.

### 2.2. G51D Oligomers Display Marked Structural Differences, including Increased α-Helical Content and Decreased Surface Hydrophobicity

**Figure 2:**
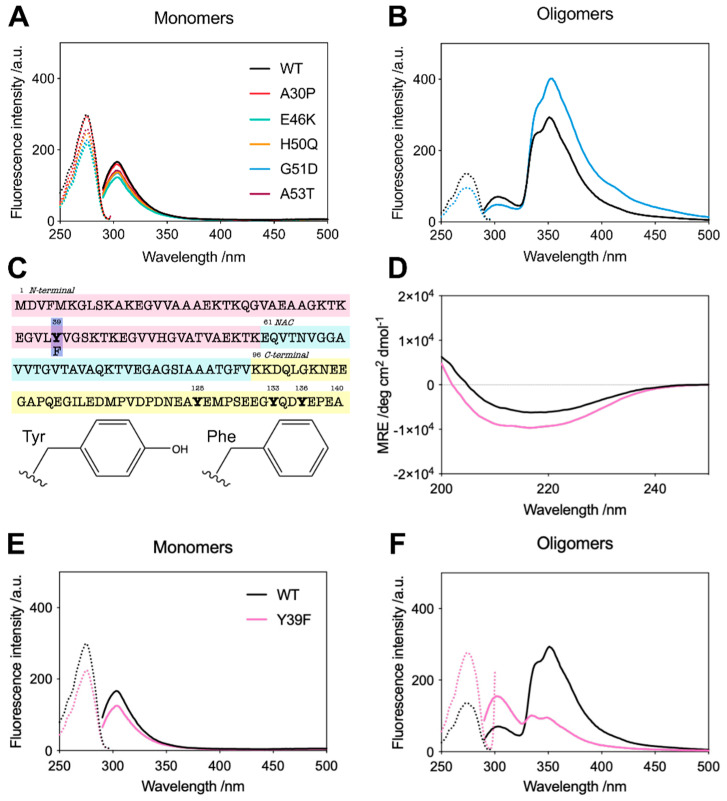
(**A**) Excitation (dotted lines) and fluorescence emission (solid lines) spectra for the monomeric α-synuclein variants. (**B**) Excitation (dotted lines) and emission (solid lines) spectra for the WT (black) and G51D (blue) oligomers. (**C**) Primary sequence of α-synuclein showing the location of Y39 and the change from Tyr-to-Phe. (**D**) CD spectra of the WT (black) and Y39F (pink) oligomers. (**E**) Excitation (dotted lines) and emission (solid lines) spectra for the WT (black) and Y39F (pink) monomers. (**F**) Excitation (dotted lines) and emission (solid lines) spectra for the WT (black) and Y39F (pink) oligomers. All spectra are representative of three independent experiments.

During the analysis of G51D oligomers, we found that G51D oligomers had a higher molar extinction coefficient (12,444 M^−1^ cm^−1^) than WT oligomers (7000 M^−1^ cm^−1^); in all variants, the oligomer extinction coefficient was higher than that of the monomeric protein (5600 M^−1^ cm^−1^). These differences were determined by comparing the UV-vis absorbance spectra with protein quantification by the following three complementary methods: amino acid analysis, bicinchoninic acid assay, and oligomer denaturation followed by SDS-PAGE to determine the monomer concentration (Figure S3). We further analysed the spectral properties of the oligomers, finding marked differences between the intrinsic tyrosine fluorescence spectra of the variants. The monomeric proteins displayed a maximum fluorescence emission peak at 305 nm, typical of tyrosine (Figure 2A). However, both the WT and G51D oligomers displayed a maximum emission peak around 345 nm, suggesting the formation of tyrosinate in the excited state [46,47]. The intensity of this 345 nm peak is much stronger in G51D oligomers while the tyrosine emission at 305 nm is less intense compared to WT oligomers, suggesting a stronger stabilisation of the tyrosinate form in the excited state in the G51D oligomers than in the WT protein, which explains the increased extinction coefficient observed for G51D oligomers (Figure 2B) [46,47].

α-Synuclein contains 4 tyrosine residues, located at positions 39, 125, 133, and 136 (Figure 2C). The latter three, located in the C-terminus of the protein, are unlikely to be involved in structural rearrangements as this region remains unstructured in both the membrane-bound α-helical conformation and the aggregated oligomer and fibrillar forms [13]. Y39 is thus the only tyrosine residue that is likely to be in a different local environment in the monomeric and oligomeric species, so we employed the Y39F variant to investigate its role in the spectral properties of the oligomers. We compared the circular dichroism (CD) spectra of the Y39 oligomers to the WT oligomers (Figure 2D), confirming their similar secondary structure, and then measured the intrinsic fluorescence properties of the monomeric and oligomeric forms of this variant (Figure 2E,F). As predicted, Y39F oligomers display tyrosine fluorescence emission at 305 nm and they lack the strong 345 nm emission that is present in the WT oligomers and accentuated in the G51D oligomers, suggesting structural differences between the monomeric and oligomeric conformations in the N-terminal region of the protein, around position 39, which are stronger in the case of the G51D pathological variant. Overall, these data highlight the structural differences between G51D oligomers and the oligomers from the other variants.

**Figure 3:**
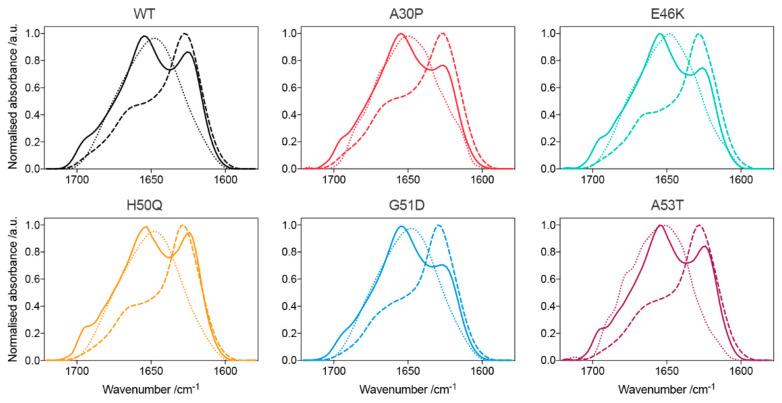
FTIR spectra of the variant α-synuclein species (solid-line: oligomers; dotted-line: monomers; dashed-line: fibrils). Peaks around 1695 cm^−1^ are characteristic of an antiparallel β-sheet structure, observed only in the oligomeric samples. Monomers display spectra typical of a random coil structure, whereas fibrils exhibit a significant degree of intermolecular β-sheet structure (peak around 1625 cm^−1^), with approximately double the β-sheet content of the oligomeric species.

**Figure 4:**
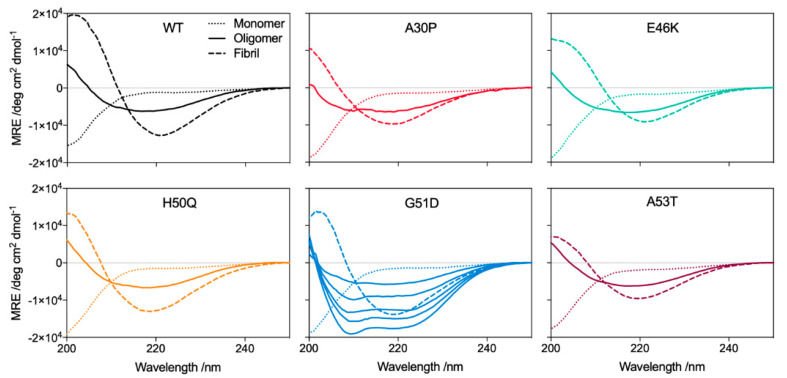
Representative (*n* > 5) CD spectra of the α-synuclein variants (solid line: oligomers; dotted line: monomers; dashed line: fibrils). All variant oligomers display a β-sheet content intermediate between that of the respective monomers and fibrils. Several G51D oligomer preparations are shown indicating the presence of a variable helical content.

Further analysis of the oligomers by Fourier transform infrared (FTIR) and CD spectroscopies revealed that all the oligomers contain a β-sheet structure, intermediate between that of their respective disordered monomeric and β-sheet-rich fibrillar states (Figure 3 and Figure 4). The FTIR spectra revealed that all oligomers contain an antiparallel β-sheet structure, in contrast to the dominant parallel β-sheet structure of the fibrils, as previously noted [45,48]. Remarkably, for the G51D oligomer preparations, we observed differing amounts of additional α-helical content by CD spectroscopy, despite all oligomers being prepared under identical conditions (Figure 4).

**Figure 5:**
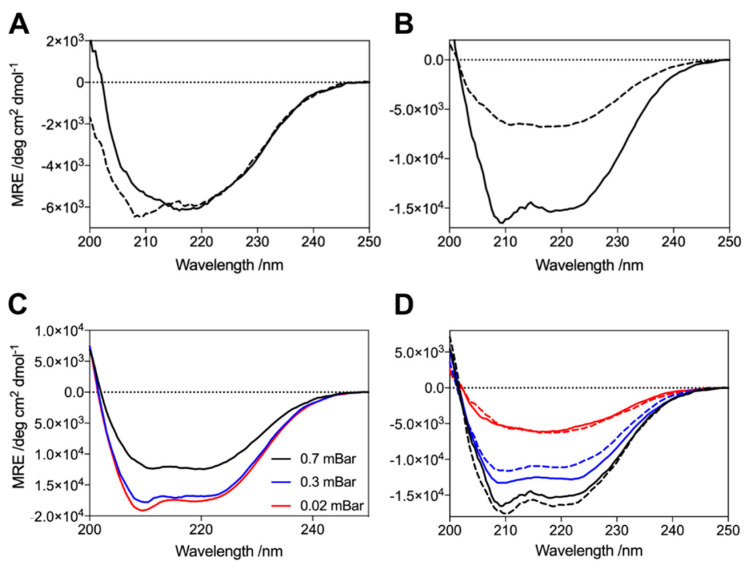
Investigation of the origins of the α-helical content of the G51D oligomers. (**A**,**B**) Oligomers produced from the flow-throughs of previous oligomer preparations do not present the same structure as the previous oligomers. Two examples are shown comparing the CD spectra of the first oligomers (solid lines) with the second oligomers (dashed). (**C**) Lyophiliser vacuum pump pressure does not dictate oligomer structure. The same monomer purification batch was lyophilised at three different vacuum pump pressures; all resulting oligomers contained α-helical structure, with no clear dependence on pressure. (**D**) Oligomer structure is determined during lyophilisation. Spectra are shown for three different preparations of lyophilised monomer (red, blue, black). Half of each batch was prepared immediately after lyophilisation (solid lines) and the other half stored at −20 °C prior to oligomer preparation on a later day (dashed lines). In each case, the structures of oligomers produced from the same lyophilised monomer stock were almost identical.

We explored the possible experimental factors that may have influenced this heterogeneity in the G51D oligomer structure. This structural variation was not due to monomer modification, for example, by oxidation or the presence of strongly bound metal ions or detergents, since oligomers successively produced from the same monomer source yielded different structures (Figure 5A,B). We next investigated the lyophilisation process, during which oligomers are formed [48]. Lyophiliser vacuum pump pressures ranging between 0.03 and 0.7 mBar led to the formation of oligomers that all contained α-helical content, indicating that the pump pressure is not sufficient to determine oligomer structure (Figure 5C). However, the preparation of oligomers on different occasions from aliquots that were lyophilised together but stored at −20 °C for different lengths of time resulted in oligomers with identical structures, demonstrating that oligomer structure is determined during lyophilisation (Figure 5D). Despite all the other α-synuclein protein variants being treated identically, only G51D formed oligomers with significant α-helical content. The distinct structural polymorphism in G51D oligomers, therefore, appears to arise from inherent differences in the aggregation process under limited hydration conditions [48].

**Figure 6:**
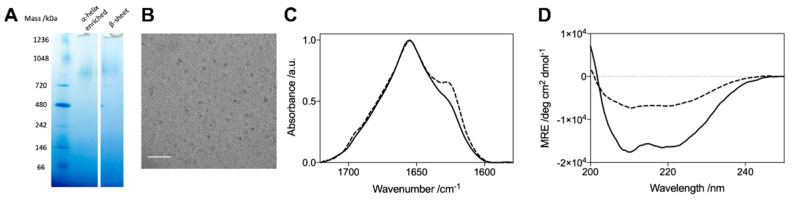
Comparison of G51D oligomers with a varying α-helical content by (**A**) Native-PAGE stained with Coomassie blue; (**B**) TEM image of the G51D oligomers with an increased helical content (scale bar = 100 nm); representative FTIR (**C**) and CD (**D**) spectra of G51D oligomers with increased α-helicity (solid line) and G51D oligomers with very little α-helical content (dashed line).

Although the different G51D oligomer preparations gave rise to distinctive α-helical spectra in the CD analysis, their corresponding FTIR spectra were comparable. The FTIR spectra indicate that all preparations contained a similar β-sheet content, and a similar combined content of α-helical and random coil structure. These results suggest that the variations in the α-helical content evident by CD are compensated for by concomitant changes in the random coil content such that the β-sheet content is largely unchanged, resulting in similar FTIR spectra. Furthermore, the β-sheet content of the G51D oligomers was also similar to that of the other variant oligomers (Figure 6C,D) [45]. These results suggest that the α-helical structure arises from regions that remain disordered in the WT and other variant oligomers. Despite the differences in the secondary structural content, no variations in the size and morphology of the helical G51D oligomers were observed (Figure 6A,B).

### 2.3. G51D Oligomer Polymorphs with High Helical Content Exhibit the Highest Cytotoxicity

**Figure 7:**
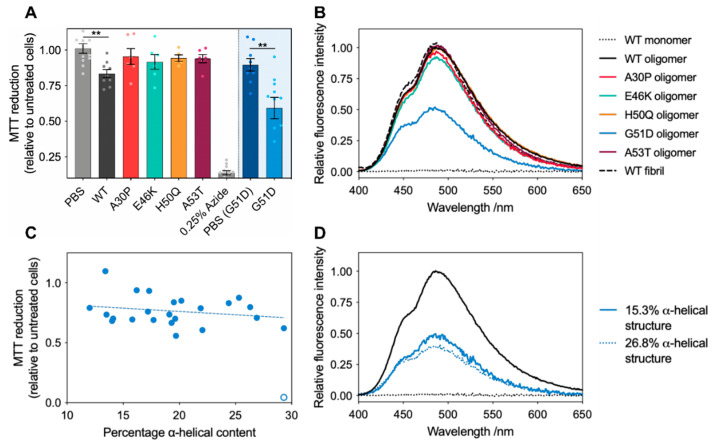
(**A**) Variant oligomer toxicity tested with the MTT assay on SH-SY5Y cells, with error bars showing the standard error (*n* ≥ 5) and data for all individual replicates overlaid as points. Due to the very low concentration yields of G51D oligomers, an additional higher volume PBS control was required, shown as PBS (G51D). Statistical analysis was run using one-way ANOVA (all variants except for G51D) or Student’s *t*-test (G51D only) ** (*p* ≤ 0.01). (**B**) Representative ANS fluorescence emission spectra of variant oligomers (*n* ≥ 3). (**C**) MTT reduction as a function of the α-helical content of G51D oligomers as estimated by far-UV CD spectra deconvolution [51,52]. The fitted linear relationship between the toxicity and percentage α-helical content in G51D oligomers is shown by the dotted line (the outlier data point (open circle) was not included in this analysis). (**D**) Representative ANS fluorescence emission spectra of WT (black) and G51D oligomers (blue) with different degrees of α-helical structure.

With this set of oligomeric species, we sought to investigate their toxicity towards cells using the MTT test, an indicator of cellular stress [49]. We previously investigated the toxicity of WT oligomers to SH-SY5Y cells, and the MTT reduction to 83% (standard deviation = 8%) reported here is in good agreement with previous studies [50]. Surprisingly, unlike the A30P, E46K, H50Q, and A53T variant oligomers that showed insignificant MTT reduction effects under the experimental conditions used, G51D oligomers displayed significantly higher toxicity than the WT oligomers (Figure 7A).

Oligomer toxicity has been shown to be largely dependent on high surface hydrophobicity, proposed to increase oligomer toxicity by increasing the oligomers’ affinity for the membrane interior via non-specific hydrophobic interactions, thus facilitating membrane disruption and cellular dysfunction [53,54,55]. We therefore investigated if this was a key factor contributing to the increased toxicity observed for the G51D oligomers by measuring the interactions of the variant oligomers with 8-anilino-naphthalene sulphate (ANS), a dye whose fluorescence emission is enhanced upon binding to solvent-exposed hydrophobic regions in the proteins [54,56]. Interestingly, whereas A30P, E46K, H50Q, and A53T oligomers showed similar solvent-accessible hydrophobicity to the WT oligomers, the oligomers generated by the G51D variant exhibited a significantly reduced hydrophobic surface (Figure 7B). Based on these studies, it would therefore be expected that G51D would be the least toxic of all the α-synuclein variants. Furthermore, as all variant oligomers exhibited the same size range, the previously identified toxicity determinants of small size and high hydrophobicity were clearly not sufficient to explain the dramatically higher cytotoxicity of the G51D oligomers [56].

Given that the variation in cellular dysfunction between experiments for the G51D oligomers was higher than any of the other variants, and the observation that G51D oligomers can have variable degrees of α-helical content, we probed further whether the increased variance in the measured cell toxicity may be linked to this observed structural polymorphism. In order to explore whether the variation in the α-helical content of G51D oligomers correlates with changes in cellular dysfunction, we characterised structurally distinct G51D oligomers. By deconvoluting the CD spectra, we were able to estimate the relative secondary structural content of oligomer preparations [51,52]. These fits reproduced our experimental data with extremely low residuals, indicating that this is a robust method for comparatively analysing our spectra (Figure S4). Deconvolution of CD spectra of WT oligomers suggests that they contain around 11% of α-helical structure, which was not detected by solid-state NMR analysis [13], indicating that the percentage of α-helical content we report here should only be used as a relative quantification between oligomer samples.

Having already observed that the size and morphology of G51D oligomers do not vary with changes in their secondary structure (Figure 6), we additionally confirmed that preparations with different degrees of α-helical structure yielded almost identical hydrophobicity readouts by ANS (Figure 7D). Furthermore, the WT and G51D oligomers were detected by the A11 antibody (proposed to bind toxic oligomers) with similar affinities (Figure S6) [57]. We thus found that the secondary structure content is the only significant structural and morphological difference between these G51D oligomer preparations. Even considering the large variability observed in the MTT assay measurements (Figure 7A), upon testing the toxicities of these distinct G51D oligomers, we identified a clear trend between an increased α-helical content and increased cellular dysfunction (Figure 7C). However, no correlation was observed between the cell toxicity and level of β-sheet or random coil structures, suggesting that the variations in cellular dysfunction can be primarily attributed to the changes in the α-helical content (Figure S5). These results thus suggest that α-helical content constitutes an additional determinant of α-synuclein oligomer toxicity.

Indeed, α-helical content may be particularly significant in the context of the aggregation and toxicity of α-synuclein, given its propensity to form highly α-helix-rich structures upon binding to lipids, which is believed to be the first requirement for triggering oligomer-mediated cell damage [58]. Moreover, α-helical content has previously been observed during the aggregation of several α-synuclein variants [59,60].

Detailed work on the WT oligomers generated through our methods has identified the mechanistic features of α-synuclein oligomer-induced membrane disruption: first, the disordered N-terminal regions of the oligomers were found to act as anchors to the membrane by folding into α-helices, allowing the structured hydrophobic β-sheet core of the oligomer to insert into the interior of the lipid bilayer [13]. The membrane anchoring step, therefore, seems to be critical for the induction of toxicity of α-synuclein oligomers. In our study, despite the lower hydrophobic nature of the G51D oligomers, we observed an enhanced cellular toxicity, which is correlated with an increased degree of α-helical structure, relative to the WT oligomers, which, according to our tyrosinate fluorescence emission analysis, is likely to occur in the N-terminal region of the protein, close to residue Y39. This may suggest that G51D oligomers have a significant pre-formed helical structure in the N-terminal region of the protein that facilitates more efficient anchoring of the oligomers to the membranes. This may allow for a more efficient insertion of the hydrophobic β-sheet core into the lipid bilayer, thus causing membrane disruption, or alternatively, the induction of other cellular dysfunction mechanisms triggered at the plasmatic membrane level that do not involve membrane bilayer perturbation. In support of the latter toxicity mechanism, G51D oligomers have previously been reported to present a much reduced membrane disruption ability relative to other variants [33]. Regardless of the specific mechanisms involved, our results indicate that amyloid oligomer toxicity may not be solely determined by oligomer surface hydrophobicity but is likely to also be dependent on other structural features of the oligomers.

## 3. Materials and Methods

### 3.1. Preparation of Oligomers

Oligomers were prepared as previously described [45]. Briefly, α-synuclein was purified into PBS [61], and subsequently dialysed against water (4 L) (ON, 4 °C). In total, 6 mg aliquots were lyophilised (48 h), followed by resuspension in buffer (500 μL PBS). The resuspended protein was passed through 0.22 μm filters and incubated (20–24 h, 37 °C). The samples were ultracentrifuged (1 h, 288,000 rcf, 20 °C) in a TLA 120.2 rotor, using an Optima TLX Ultracentrifuge (both Beckman Coulter, High Wycombe, UK) to remove aggregates and large oligomers. Remaining monomer was removed using a 100 kDa centrifugation filter (4 × 2 min, 9300 rcf). The flow-through containing predominantly monomer from the first three passes was kept and reused up to five times. The oligomer concentration was determined using UV-vis spectroscopy, using molar extinction coefficients of 7000 M^−1^ cm^−1^ for WT, E46K, H50Q, and A53T, and 12,444 M^−1^ cm^−1^ for A30P and G51D, with molar extinction coefficients determined using amino acid analysis and BCA assays.

### 3.2. Bicinchoninic Acid Assay

Bicinchoninic acid (BCA) assays were performed using a kit and bovine serum albumin (BSA) (both Thermo Scientific, Rockford, IL, USA), and carried out in Corning 96 well plates (3635). In total, 200 μL of working reagent were added to 25 μL of sample, and incubated at 37 °C for 45 min. Following incubation, the absorbance at 562 nm of each sample was recorded on a FLUOstar Optima plate reader (BMG Labtech, Aylesbury, UK). A standard curve was generated using concentrations of stock BSA between 0 and 250 μg mL^−1^, which was used to determine the protein concentrations in the sample. In order to account for potential differences in the behaviour of BSA and α-synuclein in the assay, samples were normalised to a known α-synuclein standard sample.

### 3.3. ANS Binding

8-Anilino-1-sulfonic acid (ANS) was added to samples (5 μM protein) to a final concentration of 250 μM and subsequently incubated (30 min, 20 °C). Fluorescence emission spectra were recorded between 400 and 650 nm with an excitation wavelength of 350 nm, using a Cary Eclipse Fluorescence spectrophotometer (Agilent, Santa Clara, CA, USA).

### 3.4. Circular Dichroism Spectroscopy

α-Synuclein far-UV spectra were recorded in a quartz cuvette (1 mm path length), on a JASCO J-810 equipped with a Peltier thermal-controlled cuvette holder (Jasco (UK) Ltd., Dunmow, UK) at 20 °C. In total, 15–30 spectra were averaged and recorded between 250 and 200 nm, with a data pitch of 0.5 nm, bandwidth of 1 nm, scanning speed of 50 nm/min, and response time of 4 s. Spectra were deconvoluted using the BestSel web server [51,52].

### 3.5. Dot Blot Analysis

In total, 1 μg of monomer or oligomer was deposited onto a 0.2 μm PVDF membrane (Millipore (UK) Ltd., West Lothian, UK) and left to dry at room temperature (RT). The membranes were then blocked (5% (*w*/*v*) BSA in PBS, 1 h, RT) and subsequently incubated with A11 or 211 antibody in 5% BSA in PBS (overnight, 4 °C) at 1:5000 and 1:2000 dilutions, respectively. Membranes were washed in PBS + 0.01% Tween-20 (PBST) (3 × 10 min, RT) then incubated with secondary antibody (Alexa Fluor-488 goat anti-mouse for A11, and Alexa Fluor-488 goat anti-rabbit for 211, both 1:5000 (Thermo Fisher Scientific, Waltham, MA, USA)) in PBST (1 h, RT). Following washing in PBST (3 × 10 min, RT), membranes were imaged on a Typhoon Trio scanner and the images analysed using ImageQuant TL v2005 (both Amersham Bioscience, Little Chalfont, UK).

### 3.6. FTIR Spectroscopy

FTIR measurements were performed on a Vertex 70 (Bruker, Billerica, MA, USA), fitted with a Platinum ATR (Diamond F) (oligomer and fibril measurements) or BioATRCell II (Bruker, Billerica, MA, USA). A 2–15 μM oligomer sample (2 μL) was deposited onto the detector and dried, followed by washing with milliQ water. In total, 5 spectra, each averaged over 128 scans, with atmospheric compensation and background correction, were recorded per sample. Recorded spectra were baseline corrected in the 1720–1580 cm^−1^ (amide I) region, and normalised.

### 3.7. Intrinsic Fluorescence Spectroscopy

Intrinsic fluorescence spectra were recorded on a Cary Eclipse fluorescence spectrophotometer. For emission spectra, samples were excited at 276 nm while for excitation spectra, emission was monitored at 305 nm.

### 3.8. MTT Cell Viability Assay

Human SH-SY5Y neuroblastoma cells (A.T.C.C., Manassas, VA, USA) were cultured in 1:1 Dulbecco’s modified Eagle’s medium (DMEM)-F12+GlutaMax supplement (Thermo Fisher Scientific, Waltham, MA, USA) supplemented with 10% foetal bovine serum. The cells were maintained in a 5.0% CO_2_ humidified atmosphere at 37 °C and grown to 80% confluence for a maximum of 20 passages. SH-SY5Y cells were plated in a 96-well plate at a concentration of 10,000 cells/well and treated for 24 h at 37 °C with the different α-synuclein species. After this, the cells were incubated with 0.5 mg/mL MTT 23 (3-(4,5-dimethylthiazol-2-yl)-2,5-diphenyltetrazolium bromide) in RPMI (Thermo Fisher Scientific, Waltham, MA, USA) solution at 37 °C for 4 h and subsequently lysed with a solution of 20% SDS, 50% N,N-dimethylformamide, pH 4.7 at 37 °C for 2 h. Absorbance values of blue formazan were determined at 590 nm using a Clariostar plate reader (BMG Labtech, Aylesbury, UK). We further verified that the observed differences in the MTT reduction between the α-synuclein samples was not due to LPS contamination (Figure S7).

### 3.9. Native Polyacrylamide Gel Electrophoresis

Samples were mixed with 4X loading buffer and run on NativePAGE^TM^ 4–16% Bis-Tris gels (Thermo Scientific, Rockford, IL, USA), alongside NativeMark^TM^ Unstained Protein Standard (Invitrogen by Thermo Fisher Scientific, Carlsbad, CA, USA), for 105 min at 125 V using NativePAGE^TM^ Cathode Buffer Additive and NativePAGE^TM^ Running Buffer (both Novex, Carlsbad, CA, USA). Gels were destained with a mixture of water, ethanol, and acetic acid (5:4:1 volume ratio).

### 3.10. Transmission Electron Microscopy

Each sample (10 μM, 10 μL) was adsorbed onto carbon-coated 400 mesh, 3 mm copper grids (EM Resolutions, Saffron Walden, UK). Once dry, grids were washed (2 × 10 μL water), followed by staining with 2% (*w*/*v*) uranyl acetate, and further washes (2 × 5 μL water). The samples were imaged on a FEI Tecnai G2 transmission electron microscope operating at 80 kV (Cambridge Advanced Imaging Centre (CAIC), University of Cambridge, Cambridge, UK). Images were analysed using the SIS Megaview II Image Capture system.

## 4. Conclusions

In conclusion, our findings demonstrate that the structure of stable oligomers formed from a number of mutational variants (A30P, E46K, A53T, H50Q) are very similar to the WT protein; however, distinct structural polymorphs are produced from the G51D variant. Despite the decreased hydrophobicity within the G51D oligomers, they display more cytotoxicity than the other variant oligomers. Previous extensive experimental work has established size and hydrophobicity as the key molecular determinants for oligomer toxicity [7,44,56,62]; our results indicate that additional oligomer structure–toxicity relationships may exist. The ability to study the polymorphic G51D oligomers allowed us to show that the α-helical content is a good predictor of their cytotoxicity and thus constitutes an additional structural attribute that correlates with cellular dysfunction. Secondary structure polymorphism in fibrils has been studied in detail in the context of strains and toxicity [63,64,65]. Although the vast majority of such structures consist predominantly of the canonical amyloid β-sheet structure, recent work has identified the α-helical content as a key structural element in fibrils of the most toxic member of the phenol-soluble modulin (PSM) family, with toxicity being clearly linked to this secondary structure element [66,67]. In light of the profound effect of small structural changes on the properties of fibrils, the significant intrinsic heterogeneity of oligomer structures, in particular our findings of the G51D oligomers, may be a key determinant of their aggregation propensity and toxicity.

## Data Availability

Not applicable.

## Supplementary Materials

The following supporting information can be downloaded online. Figure S1 (primary structure of α-synuclein and native-PAGE analysis of oligomers); Figure S2 (dynamic light scattering analysis of variant monomers and oligomers); Figure S3 (assays to confirm extinction coefficients of oligomer species); Figure S4 (BestSel fitting of experimental CD data); Figure S5 (analysis of the relationship between G51D oligomer secondary structure and MTT reduction); Figure S6 (dot blot analysis of WT and G51D oligomers with conformational specific antibody A11); Figure S7 (quantification of LPS in α-synuclein stocks).

## Sample Availability

DNA plasmids are available from the authors.

## References

[1] Chiti F., Dobson C.M. (2017). Protein misfolding, amyloid formation, and human disease: A summary of progress over the last decade. Annu. Rev. Biochem..

[2] Fusco G., De Simone A., Gopinath T., Vostrikov V., Vendruscolo M., Dobson C.M., Veglia G. (2014). Direct observation of the three regions in alpha-synuclein that determine its membrane-bound behaviour. Nat. Comm..

[3] Snead D., Eliezer D. (2014). Alpha-synuclein function and dysfunction on cellular membranes. Exp. Neurobiol..

[4] Auluck P.K., Caraveo G., Lindquist S. (2010). α-Synuclein: Membrane interactions and toxicity in Parkinson’s disease. Annu. Rev. Cell Dev. Biol..

[5] Butterfield S.M., Lashuel H.A. (2010). Amyloidogenic protein-membrane interactions: Mechanistic insight from model systems. Angew. Chem. Int. Ed..

[6] Galvagnion C., Buell A.K., Meisl G., Michaels T.C.T., Vendruscolo M., Knowles T.P.J., Dobson C.M. (2015). Lipid vesicles trigger α-synuclein aggregation by stimulating primary nucleation. Nat. Chem. Biol..

[7] Cremades N., Cohen S.I.A., Deas E., Abramov A.Y., Chen A.Y., Orte A., Sandal M., Clarke R.W., Dunne P., Aprile F.A. (2012). Direct observation of the interconversion of normal and toxic forms of alpha-synuclein. Cell.

[8] Bemporad F., Chiti F. (2012). Protein misfolded oligomers: Experimental approaches, mechanism of formation, and structure-toxicity relationships. Chem. Biol..

[9] Glabe C.G. (2006). Common mechanisms of amyloid oligomer pathogenesis in degenerative disease. Neurobiol. Aging.

[10] Hartl F.U. (2017). Protein misfolding diseases. Annu Rev. Biochem.

[11] Giampà M., Amundarain M.J., Herrera M.G., Tonali N., Dodero V.I. (2021). Implementing complementary approaches to shape the mechanism of α-synuclein oligomerization as a model of amyloid aggregation. Molecules.

[12] Andreasen M., Lorenzen N., Otzen D. (2015). Interactions between misfolded protein oligomers and membranes: A central topic in neurodegenerative diseases?. Biochim. Biophys. Acta.

[13] Fusco G., Chen S.W., Williamson P.T.F., Cascella R., Perni M., Jarvis J.A., Cecchi C., Vendruscolo M., Chiti F., Cremades N. (2017). Structural basis of membrane disruption and cellular toxicity by α-synuclein oligomers. Science.

[14] Musteikyte G., Jayaram A.K., Xu C.K., Vendruscolo M., Krainer G., Knowles T.P.J. (2021). Interactions of alpha-synuclein oligomers with lipid membranes. Biochim. Biophys. Acta Biomembr..

[15] Spillantini M.G., Schmidt M.L., Lee V.M., Trojanowski J.Q., Jakes R., Goedert M. (1997). Alpha-synuclein in Lewy bodies. Nature.

[16] Conway K.A., Lee S.J., Rochet J.C., Ding T.T., Williamson R.E., Lansbury P.T. (2000). Acceleration of oligomerization, not fibrillization, is a shared property of both alpha-synuclein mutations linked to early-onset Parkinson’s disease: Implications for pathogenesis and therapy. Proc. Natl. Acad. Sci. USA.

[17] Flagmeier P., Meisl G., Vendruscolo M., Knowles T.P.J., Dobson C.M., Buell A.K., Galvagnion C. (2016). Mutations associated with familial Parkinson’s disease alter the initiation and amplification steps of α-synuclein aggregation. Proc. Natl. Acad. Sci. USA.

[18] Fredenburg R.A., Rospigliosi C., Meray R.K., Kessler J.C., Lashuel H.A., Eliezer D., Lansbury P.T. (2007). The impact of the E46K mutation on the properties of α-synuclein in its monomeric and oligomeric states. Biochemistry.

[19] Khalaf O., Fauvet B., Oueslati A., Dikiy I., Mahul-Mellier A.-L., Ruggeri F.S., Mbefo M.K., Vercruysse F., Dietler G., Lee S.-J. (2014). The H50Q mutation enhances α-synuclein aggregation, secretion, and toxicity. J. Biol. Chem..

[20] Lashuel H.A., Hartley D., Petre B.M., Walz T., Lansbury P.T. (2002). Neurodegenerative disease: Amyloid pores from pathogenic mutations. Nature.

[21] Lemkau L.R., Comellas G., Kloepper K.D., Woods W.S., George J.M., Rienstra C.M. (2012). Mutant protein A30P α-synuclein adopts wild-type fibril structure, despite slower fibrillation kinetics. J. Biol. Chem..

[22] Li J., Uversky V.N., Fink A.L. (2001). Effect of familial Parkinson’s disease point mutations A30P and A53T on the structural properties, aggregation, and fibrillation of human α-synuclein. Biochemistry.

[23] Narhi L., Wood S.J., Steavenson S., Jiang Y., Wu G.M., Anafi D., Kaufman S.A., Martin F., Sitney K., Denis P. (1999). Both familial Parkinson’s disease mutations accelerate alpha-synuclein aggregation. J. Biol. Chem..

[24] Rutherford N.J., Moore B.D., Golde T.E., Giasson B.I. (2014). Divergent effects of the H50Q and G51D SNCA mutations on the aggregation of alpha-synuclein. J. Neurochem..

[25] Bodner C.R., Maltsev A.S., Dobson C.M., Bax A. (2010). Differential phospholipid binding of alpha-synuclein variants implicated in Parkinson’s disease revealed by solution NMR spectroscopy. Biochemistry.

[26] Bussell R., Eliezer D. (2004). Effects of Parkinson’s disease-linked mutations on the structure of lipid-associated α-synuclein. Biochemistry.

[27] Choi W., Zibaee S., Jakes R., Serpell L.C., Davletov B., Anthony Crowther R., Goedert M. (2004). Mutation E46K increases phospholipid binding and assembly into filaments of human α-synuclein. FEBS Lett..

[28] Fusco G., De Simone A., Arosio P., Vendruscolo M., Veglia G., Dobson C.M. (2016). Structural ensembles of membrane-bound α-synuclein reveal the molecular determinants of synaptic vesicle affinity. Sci. Rep..

[29] Jensen P.H., Nielsen M.S., Jakes R., Dotti C.G., Goedert M. (1998). Binding of α-synuclein to brain vesicles is abolished by familial Parkinson’s disease mutation. J. Biol. Chem..

[30] Jo E., Fuller N., Rand R.P., St George-Hyslop P., Fraser P.E. (2002). Defective membrane interactions of familial Parkinson’s disease mutant A30P α-synuclein. J. Mol. Biol..

[31] Jo E., McLaurin J., Yip C.M., St George-Hyslop P., Fraser P.E. (2000). α-synuclein membrane interactions and lipid specificity. J. Biol. Chem..

[32] Perrin R.J., Woods W.S., Clayton D.F., George J.M. (2000). Interaction of human α-synuclein and Parkinson’s disease variants with phospholipids: Structural analysis using site-directed mutagenesis. J. Biol. Chem..

[33] Stefanovic A.N.D., Lindhoud S., Semerdzhiev S.A., Claessens M.M.A.E., Subramaniam V. (2015). Oligomers of Parkinson’s disease-related α-synuclein mutants have similar structures but distinctive membrane permeabilization properties. Biochemistry.

[34] Ysselstein D., Joshi M., Mishra V., Griggs A.M., Asiago J.M., McCabe G.P., Stanciu L.A., Post C.B., Rochet J.-C. (2015). Effects of impaired membrane interactions on α-synuclein aggregation and neurotoxicity. Neurobiol. Dis..

[35] Middleton E.R., Rhoades E. (2010). Effects of curvature and composition on α-synuclein binding to lipid vesicles. Biophys. J..

[36] Fares M.-B., Ait-Bouziad N., Dikiy I., Mbefo M.K., Jovicic A., Kiely A., Holton J.L., Lee S.-J., Gitler A.D., Eliezer D. (2014). The novel Parkinson’s disease linked mutation G51D attenuates in vitro aggregation and membrane binding of α-synuclein, and enhances its secretion and nuclear localization in cells. Hum. Mol. Genet..

[37] Ghosh D., Mondal M., Mohite G.M., Singh P.K., Ranjan P., Anoop A., Ghosh S., Jha N.N., Kumar A., Maji S.K. (2013). The Parkinson’s disease-associated H50Q mutation accelerates α-synuclein aggregation in vitro. Biochemistry.

[38] Giasson B.I., Uryu K., Trojanowski J.Q., Lee V.M.Y. (1999). Mutant and wild type human α-synucleins assemble into elongated filaments with distinct morphologies in vitro. J. Biol. Chem..

[39] Li J., Uversky V.N., Fink A.L. (2002). Conformational behavior of human α-synuclein is modulated by familial Parkinson’s disease point mutations A30P and A53T. Neurotoxicology.

[40] Bucciantini M., Giannoni E., Chiti F., Baroni F., Formigli L., Zurdo J., Taddei N., Ramponi G., Dobson C.M., Stefani M. (2002). Inherent toxicity of aggregates implies a common mechanism for protein misfolding diseases. Nature.

[41] Chiti F., Dobson C.M. (2006). Protein misfolding, functional amyloid, and human disease. Annu. Rev. Biochem..

[42] Stefani M., Dobson C.M. (2003). Protein aggregation and aggregate toxicity: New insights into protein folding, misfolding diseases and biological evolution. J. Mol. Med..

[43] Paslawski W., Mysling S., Thomsen K., Jørgensen T.J.D., Otzen D.E. (2014). Co-existence of two different α-synuclein oligomers with different core structures determined by hydrogen/deuterium exchange mass spectrometry. Angew. Chem. Int. Ed..

[44] Tosatto L., Horrocks M.H., Dear A.J., Knowles T.P.J., Dalla Serra M., Cremades N., Dobson C.M., Klenerman D. (2015). Single-molecule FRET studies on alpha-synuclein oligomerization of Parkinson’s disease genetically related mutants. Sci. Rep..

[45] Chen S.W., Drakulic S., Deas E., Ouberai M., Aprile F.A., Arranz R., Ness S., Roodveldt C., Guilliams T., De-Genst E.J. (2015). Structural characterization of toxic oligomers that are kinetically trapped during α-synuclein fibril formation. Proc. Natl. Acad. Sci. USA.

[46] Prendergast F.G., Hampton P.D., Jones B. (1984). Characteristics of tyrosinate fluorescence emission in α and β-purothionins. Biochemistry.

[47] Szabo A.G., Lynn K.R., Krajcarski D.T., Rayner D.M. (1978). Tyrosinate fluorescence maxima at 345 nm in proteins lacking tryptophan at pH 7. FEBS Lett..

[48] Camino J.D., Gracia P., Chen S.W., Sot J., de la Arada I., Sebastian V., Arrondo J.L.R., Goni F.M., Dobson C.M., Cremades N. (2020). The extent of protein hydration dictates the preference for heterogeneous or homogeneous nucleation generating either parallel or antiparallel beta-sheet alpha-synuclein aggregates. Chem. Sci..

[49] Mosmann T. (1983). Rapid colorimetric assay for cellular growth and survival: Application to proliferation and cytotoxicity assays. J. Immunol. Methods.

[50] Perni M., Galvagnion C., Maltsev A., Meisl G., Müller M.B.D., Challa P.K., Kirkegaard J.B., Flagmeier P., Cohen S.I.A., Cascella R. (2017). A natural product inhibits the initiation of alpha-synuclein aggregation and suppresses its toxicity. Proc. Natl. Acad. Sci. USA.

[51] Micsonai A., Wien F., Bulyáki É., Kun J., Moussong É., Lee Y.-H., Goto Y., Réfrégiers M., Kardos J. (2018). BeStSel: A web server for accurate protein secondary structure prediction and fold recognition from the circular dichroism spectra. Nucleic Acids Res..

[52] Micsonai A., Wien F., Kernya L., Lee Y.-H., Goto Y., Réfrégiers M., Kardos J. (2015). Accurate secondary structure prediction and fold recognition for circular dichroism spectroscopy. Proc. Natl. Acad. Sci. USA.

[53] Bolognesi B., Kumita J.R., Barros T.P., Esbjörner E.K., Luheshi L.M., Crowther D.C., Wilson M.R., Dobson C.M., Favrin G., Yerbury J.J. (2010). ANS binding reveals common features of cytotoxic amyloid species. ACS Chem. Biol..

[54] Campioni S., Mannini B., Zampagni M., Pensalfini A., Parrini C., Evangelisti E., Relini A., Stefani M., Dobson C.M., Cecchi C. (2010). A causative link between the structure of aberrant protein oligomers and their toxicity. Nat. Chem. Biol..

[55] Oma Y., Kino Y., Toriumi K., Sasagawa N., Ishiura S. (2007). Interactions between homopolymeric amino acids (HPAAs). Protein Sci..

[56] Mannini B., Mulvihill E., Sgromo C., Cascella R., Khodarahmi R., Ramazzotti M., Dobson C.M., Cecchi C., Chiti F. (2014). Toxicity of protein oligomers is rationalized by a function combining size and surface hydrophobicity. ACS Chem. Biol..

[57] Kayed R., Head E., Sarsoza F., Saing T., Cotman C.W., Necula M., Margol L., Wu J., Breydo L., Thompson J.L. (2007). Fibril specific, conformation dependent antibodies recognize a generic epitope common to amyloid fibrils and fibrillar oligomers that is absent in prefibrillar oligomers. Mol. Neurodegener..

[58] Stefanovic A.N.D., Stöckl M.T., Claessens M.M.A.E., Subramaniam V. (2014). Alpha-synuclein oligomers distinctively permeabilize complex model membranes. FEBS J..

[59] Apetri M.M., Maiti N.C., Zagorski M.G., Carey P.R., Anderson V.E. (2006). Secondary structure of α-synuclein oligomers: Characterization by Raman and Atomic Force Microscopy. J. Mol. Biol..

[60] Ghosh D., Singh P.K., Sahay S., Jha N.N., Jacob R.S., Sen S., Kumar A., Riek R., Maji S.K. (2015). Structure based aggregation studies reveal the presence of helix-rich intermediate during α-synuclein aggregation. Sci. Rep..

[61] Hoyer W., Antony T., Cherny D., Heim G., Jovin T.M., Subramaniam V. (2002). Dependence of α-synuclein aggregate morphology on solution conditions. J. Mol. Biol..

[62] Winner B., Jappelli R., Maji S.K., Desplats P.A., Boyer L., Aigner S., Hetzer C., Loher T., Vilar M., Campioni S. (2011). In vivo demonstration that alpha-synuclein oligomers are toxic. Proc. Natl. Acad. Sci. USA.

[63] Boyer D.R., Li B., Sun C., Fan W., Sawaya M.R., Jiang L., Eisenberg D.S. (2019). Structures of fibrils formed by α-synuclein hereditary disease mutant H50Q reveal new polymorphs. Nat. Struct. Mol. Biol..

[64] Peelaerts W., Bousset L., Van der Perren A., Moskalyuk A., Pulizzi R., Giugliano M., Van den Haute C., Melki R., Baekelandt V. (2015). Alpha-synuclein strains cause distinct synucleinopathies after local and systemic administration. Nature.

[65] Riek R., Eisenberg D.S. (2016). The activities of amyloids from a structural perspective. Nature.

[66] Salinas N., Colletier J.-P., Moshe A., Landau M. (2018). Extreme amyloid polymorphism in Staphylococcus aureus virulent PSMα peptides. Nat. Commun..

[67] Tayeb-Fligelman E., Tabachnikov O., Moshe A., Goldshmidt-Tran O., Sawaya M.R., Coquelle N., Colletier J.-P., Landau M. (2017). The cytotoxic Staphylococcus aureus PSMα reveals a cross-a amyloid-like fibril. Science.

