# Supplementary material for: The Pathological G51D Mutation in Alpha-Synuclein Oligomers Confers Distinct Structural Attributes and Cellular Toxicity
Source: Molecules. 2022 Feb 15;27(4):1293. doi: 10.3390/molecules27041293 (PMC8879557; doi:10.3390/molecules27041293)
Supplement: Supplementary file 1 [file molecules-27-01293-s001.zip › molecules-1581149-supplementary.pdf]

Supplementary Material

The pathological G51D mutation in alpha-synuclein oligomers confers distinct structural attributes and cellular toxicity

Catherine K. Xu[1], Marta Castellana-Cruz[1], Serene W. Chen[2], Zhen Du[3], Georg Meisl[1], Aviad Levin[1], Benedetta Mannini[1], Laura S. Itzhaki[3], Tuomas P.J. Knowles[1,4], Christopher M. Dobson[1,‡], Nunilo Cremades[5,*] and Janet R. Kumita[3,*]

[1] Yusuf Hamied Department of Chemistry, University of Cambridge, Lensfield Road, Cambridge CB2 1EW, UK; catherine.xu@mpl.mpg.de; mc2033@cam.ac.uk; gm373@cam.ac.uk; al680@cam.ac.uk; bm475@cam.ac.uk; tpjk2@cam.ac.uk;
[2] Department of Life Sciences, Imperial College London, South Kensington Campus, London SW7 2AZ, UK; weiyan89@gmail.com;
[3] Department of Pharmacology, University of Cambridge, Tennis Court Road, Cambridge CB2 1PD, UK; zhen_du2020@163.com; lsi10@cam.ac.uk;
[4] Cavendish Laboratory, Department of Physics, University of Cambridge, JJ Thomson Avenue, Cambridge CB3 0HE, UK;
[5] Institute for Biocomputation and Physics of Complex Systems (BIFI), University of Zaragoza, Mariano Esquillor, Edificio I+D+I, 50018 Zaragoza, Spain;
‡ Dedicated to the memory of Prof. Sir Christopher Dobson FRS, who helped and inspired us over many years and who is missed greatly by all who knew him.
* Correspondence: ncc@unizar.es (NC); jrk38@cam.ac.uk (JRK)

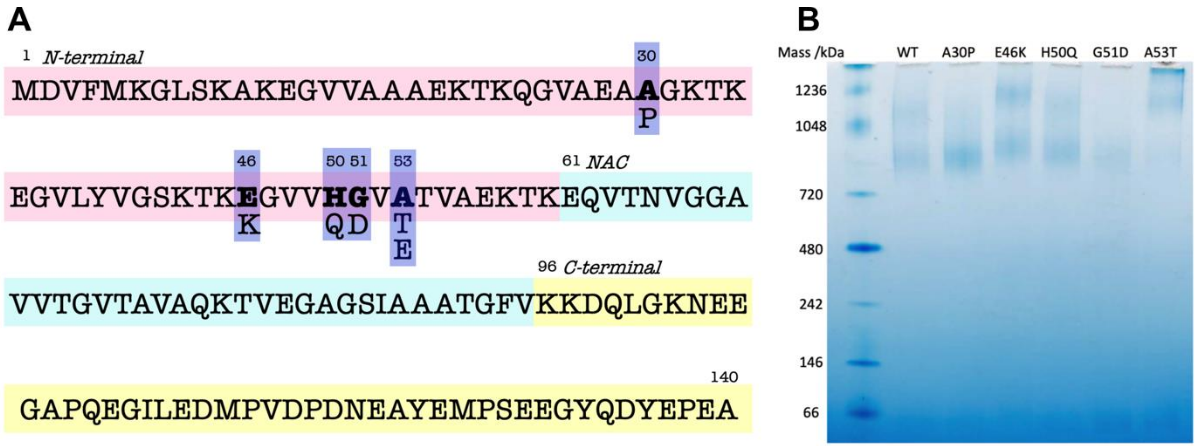

Figure S1. (**A**) Primary structure of α-synuclein, showing the locations of the disease-associated mutations (highlighted in dark blue). (**B**) Native-PAGE analysis of variant oligomers, showing the same size distributions as determined by AUC, demonstrating that native-PAGE is able to resolve oligomer size populations.

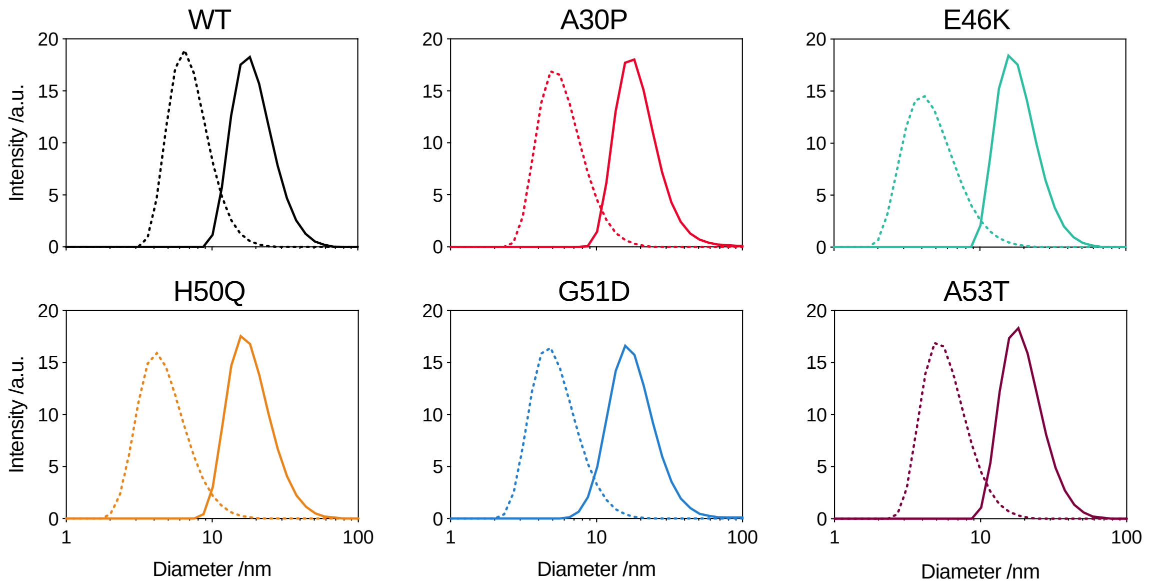

Figure S2. Dynamic light scattering (DLS) analysis of oligomers (solid) and monomers (dotted); all variants exhibit almost identical size distributions at this resolution, with monomer and oligomer populations being distinct from each other.

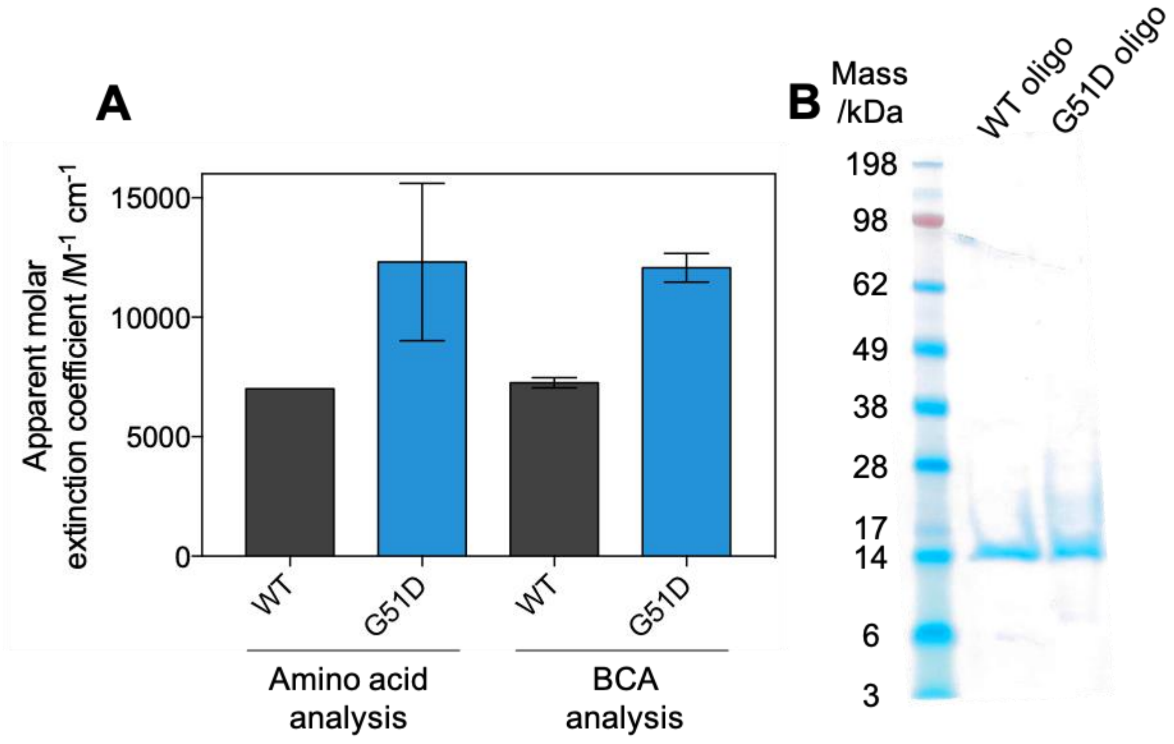

Figure S3. WT and G51D oligomers exhibit different molar extinction coefficients. **(A)** Using UV-vis spectroscopy, absorption at 275 nm of both oligomeric species was compared to protein concentration determined through amino acid analysis (WT: n = 1, G51D: n = 2) and BCA analysis (n = 4), normalised against the corresponding variant monomeric α-synuclein, and the apparent molar extinction coefficient determined (error bars for standard deviation). The two methods yielded almost identical results. We further confirmed these extinction coefficients by incubating WT and G51D oligomers, whose concentrations were determined using molar extinction coefficients of 7000 M^{-1} cm^{-1} and 12444 M^{-1} cm^{-1}, respectively, with 6 M guanidinium thiocyanate. The resulting monomeric samples were then analysed by SDS-PAGE **(B)**, and resulted in similar band intensities, indicating that these extinction coefficients are accurate.

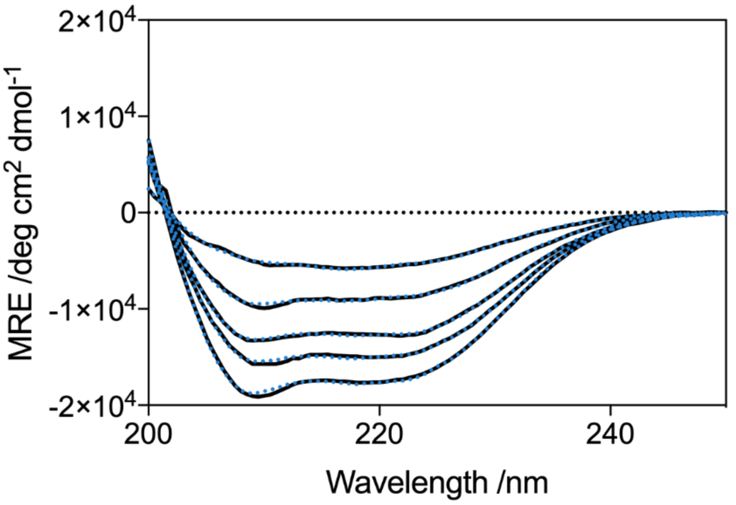

Figure S4. Fitted G51D CD spectra recapitulate the experimentally obtained spectra. CD spectra of G51D oligomers displaying different degrees of α-helical structure (black solid), shown alongside the fitted spectra (blue dotted), obtained using the BestSel software (3; 4)

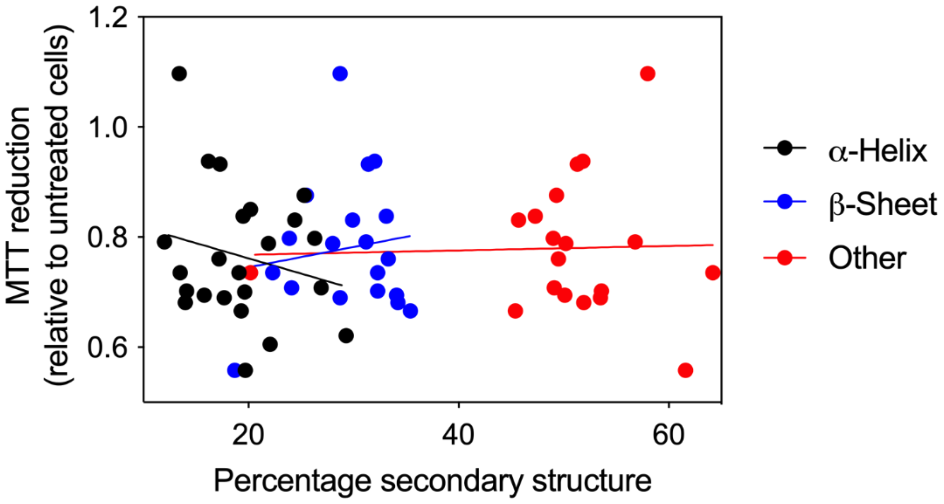

Figure S5. MTT reduction correlates only with α-helical content of oligomers; no correlation is observed with β-sheet content or random coil/other structures.

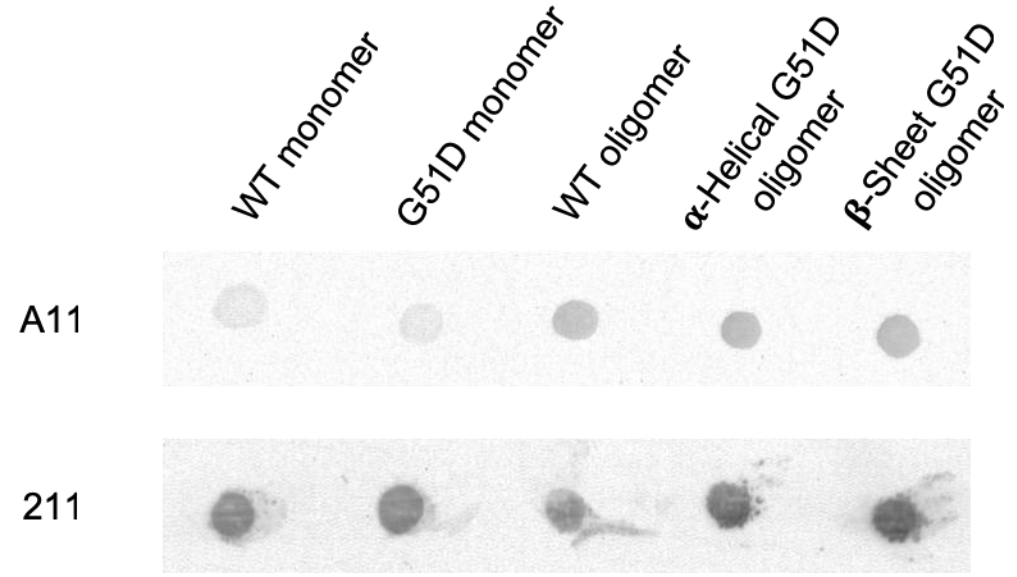

Figure S6. Both WT and isoforms of G51D oligomers bind the A11 antibody. Dot blots of WT and G51D monomers and oligomers. The A11 antibody, proposed to bind toxic amyloid oligomers, binds both WT and G51D oligomers with different conformations, with similar affinity (2). The 211 antibody has been found to bind the C-terminus of α-synuclein, and was therefore used as a control to confirm that the difference in binding of A11 to monomers and oligomers was not due to differences in α-synuclein concentration (1).

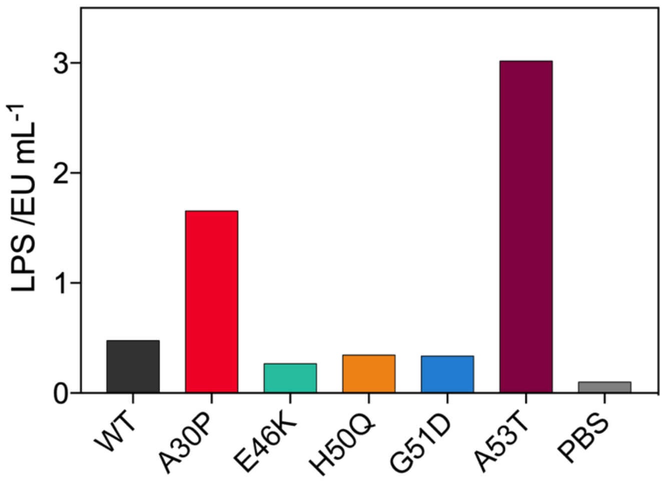

Figure S7. Quantification of LPS concentrations in α-synuclein monomer stocks. The LPS content of samples does not correlate with MTT reduction, indicating that LPS does not contribute to the observed cytotoxicity.

References:

1. Giasson BI, Uryu K, Trojanowski JQ, Lee VMY (1999) Mutant and wild type human α-synucleins assemble into elongated filaments with distinct morphologies in vitro. J Biol Chem 274:7619-7622.
2. Kayed R, Head E, Sarsoza F, Saing T, Cotman CW, Necula M, Margol L, Wu J, Breydo L, Thompson JL, Rasool S, Gurlo T, Butler P, Glabe CG (2007) Fibril specific, conformation dependent antibodies recognize a generic epitope common to amyloid fibrils and fibrillar oligomers that is absent in prefibrillar oligomers. Mol Neurodegener 2:18-18.
3. Micsonai A, Wien F, Bulyáki É, Kun J, Moussong É, Lee Y-H, Goto Y, Réfrégiers M, Kardos J (2018) BeStSel: a web server for accurate protein secondary structure prediction and fold recognition from the circular dichroism spectra. Nucleic Acids Res 46:W315-W322.
4. Micsonai A, Wien F, Kernya L, Lee Y-H, Goto Y, Réfrégiers M, Kardos J (2015) Accurate secondary structure prediction and fold recognition for circular dichroism spectroscopy. Proc Natl Acad Sci USA 112:E3095-E3103.
